# Supplementary material for: An Information-Geometric Justification for Composite Coherence in Event-Based Narrative Extraction
Source: Entropy (Basel). 2026 Jun 28;28(7):732. doi: 10.3390/e28070732 (PMC13409454; doi:10.3390/e28070732)
Supplement: Supplementary file 1 [file entropy-28-00732-s001.zip › Supplementary_Material_S1_S9.pdf]

Supplementary Materials: An Information-Geometric Justification for Composite Coherence in Event-Based Narrative Extraction

Brian Keith-Norambuena [1,*]

This document collects supporting analyses for the manuscript "An Information-Geometric Justification for Composite Coherence in Event-Based Narrative Extraction." These analyses extend the empirical validation of the main text but are not required for its central argument; they are provided here to keep the main manuscript focused. Sections 1 and 2 verify that the information-geometric properties are robust to the choice of topic model and to the cluster count. Section 3 reports the embedding-model sensitivity analysis. Section 4 gives a component ablation isolating the contribution of soft cluster membership and of the Jensen-Shannon *distance*. Section 5 gives the per-seed breakdown of the embedding-perturbation experiment (Experiment 6 of the main text). Section 6 records a heuristic rate-distortion analogy for the geometric mean. Section 7 reports a path-level bootstrap quantifying the sampling uncertainty of the Experiment 7 bottleneck-gap profile. Section 8 presents a cross-modal case study, applying the bottleneck-gap analysis to a human-curated image narrative (the ROGER corpus). Section 9 validates the geometric-mean veto property under the cardinality-constrained Narrative Maps linear program, supporting the combinator-selection discussion of the main text. Cross-references of the form "the main text" point to the main manuscript.

1. Alternative Topic Models

The theoretical results of the main text apply to any soft topic assignment, not just HDBSCAN. Any method that produces a probability vector $\hat{e}_i \in \Delta_+^{K-1}$ for each document (e.g., LDA topic proportions, soft k-means memberships, neural topic model outputs) yields a topic similarity $T = 1 - d_{\mathrm{JS}}$ with the same information-geometric properties. To verify this empirically, we replace the UMAP→HDBSCAN pipeline with three alternative topic models (LDA, soft k-means, and Gaussian Mixture Models (GMM)) at $K \in \{3, 5, 6, 12, 24\}$ on the Cuba corpus, and evaluate the same metric-level properties. Table S1 summarizes the results.

Takeaway. LDA and HDBSCAN, which produce smooth (high-entropy) membership vectors, preserve the product-manifold properties across the full K range. GMM exhibits a K-dependent trade-off: at low K ($K = 3$) its $\rho_{\mathrm{GM}} = 0.980$ approaches the LDA / soft k-means level, but with 2.0% triangle inequality violations on $1 - C_{\mathrm{GM}}$; as K increases, the violations vanish (0.01% at $K = 24$) while ρ_{GM} degrades monotonically to 0.43. The two failure modes bracket the usable GMM range to a narrow neighborhood of $K = 3$–5; LDA and HDBSCAN remain clean across the full range.

The Fisher identity holds across all topic models tested ($R \geq 0.99$, and $R \geq 0.995$ for the three alternatives), indicating that this property depends only on the JSD-Fisher connection. The GM rank correlation with $d_\times$ is near-perfect for LDA at every K tested ($\rho \geq 0.999$) and high for soft k-means ($\rho \geq 0.969$), but for GMM the K-dependence is monotonic and steep: ρ_{GM} falls from 0.980 at $K = 3$ to 0.434 at $K = 24$. The degradation occurs because Gaussian mixture posteriors at high K produce sharply peaked membership vectors that concentrate near simplex vertices, approaching the hard-assignment regime where the Fisher-Rao metric tensor degenerates (cf. the soft-membership remark of the main text). Low-K GMM posteriors are smoother and preserve ρ_{GM} at the cost of higher triangle-violation rates on $1 - C_{\mathrm{GM}}$: at $K = 3$, 2.01% of triplets violate, vs. 0.01% at $K = 24$. The

Table S1. Alternative topic models on Cuba ($n = 418$). The Fisher identity (R_{Fisher}), GM rank correlation with $d_\times$ (ρ_{GM}), triangle inequality violations, and scale complementarity ($\rho_{A,T}$, NMI; lower values indicate greater non-redundancy) are reported for each method and cluster count. The $K \in \{3, 5\}$ rows test whether smoother (lower-K) GMM posteriors preserve ρ_{GM}.

**Method**	K	R_{Fisher}	ρ_{GM}	Viol. (%)	$\rho_{A,T}$	NMI
HDBSCAN (baseline)	11	0.994	0.999	0.0	0.283	0.027
LDA	3	0.996	0.999	0.01	0.165	0.010
LDA	5	0.996	1.000	0.03	0.179	0.011
LDA	6	0.995	1.000	0.01	0.227	0.016
LDA	12	0.995	1.000	0.0	0.142	0.009
LDA	24	0.997	1.000	0.0	0.198	0.017
Soft k-means	3	1.000	0.969	0.0	0.512	0.059
Soft k-means	5	1.000	0.974	0.0	0.485	0.058
Soft k-means	6	1.000	0.975	0.0	0.536	0.071
Soft k-means	12	1.000	0.984	0.0	0.610	0.096
Soft k-means	24	1.000	0.984	0.0	0.585	0.090
GMM	3	0.997	0.980	2.01	0.215	0.027
GMM	5	0.997	0.912	1.30	0.159	0.019
GMM	6	0.997	0.838	0.78	0.161	0.023
GMM	12	0.998	0.677	0.24	0.457	0.042
GMM	24	1.000	0.434	0.01	0.378	0.037

framework's practical utility therefore requires membership distributions with sufficient entropy: models whose membership vectors have low entropy (effectively hard clustering at large K) violate the smooth-manifold assumption underlying the Fisher-Rao connection. LDA's Dirichlet prior naturally smooths the membership vectors, and HDBSCAN's density-based soft assignments achieve similar smoothness. We recommend topic models that produce smooth membership distributions (LDA, HDBSCAN) across the full K range; GMM is acceptable only at low K where its posteriors remain smooth, and even there with elevated triangle-violation rates. Scale complementarity varies across these models: LDA maintains strong non-redundancy ($\rho_{A,T} \leq 0.23$, NMI ≤ 0.017) at every K, while soft k-means shows moderate correlation ($\rho_{A,T} \approx 0.48$ to 0.61), reflecting its tendency to retain more fine-grained information.

2. Cluster-Count Sensitivity

The number of topic clusters controls the granularity of the topic component. A hyperparameter sweep varying HDBSCAN's `min_cluster_size` on both Cuba and VisPub confirms that the cluster count is the most impactful pipeline parameter. Table S2 reports the results.

Takeaway. The optimal weight w^* is centered on the equal-weight value 0.5 across all but the $K = 2$ boundary configurations, where the topic channel collapses to a binary same/different signal.

Across the sweep, w^* stays within $[0.45, 0.60]$ on both corpora, with the 95% bootstrap CIs covering 0.5 in 8 of the 13 configurations. Four of the five exceptions are the degenerate $K = 2$ configurations, and the fifth (Cuba `mcs` $= 3$, $K = 31$) misses *low* ($w^* = 0.45$); the deviations from 0.5 thus occur exactly where the cluster count departs most from the mid-range, consistent with the $\rho(K, w^*) = -0.85$ trend. Effect sizes are modest ($d \leq 0.51$) and strongest at the degenerate $K = 2$ regime. Cuba at `mcs` $= 30$ ($K = 2$) gives $w^* = 0.55$ with $d = 0.51$, and VisPub at `mcs` $= 50$ ($K = 2$) gives $w^* = 0.60$ with $d = 0.42$. Away from the $K = 2$ boundary, w^* tracks 0.5 closely on both corpora (Cuba $K > 2$: $d \leq 0.25$; VisPub $K > 2$: $d \leq 0.08$), consistent with the equal-weight structure of the geometric mean.

Table S2. Cluster count sensitivity. HDBSCAN sweep varying `min_cluster_size` on Cuba and VisPub (VisPub subsampled to 500 documents for the sweep). w^* is the optimal angular-topic weight on the weighted geometric mean $C_w = A^w T^{1-w}$ (0.5 = equal weighting; w is the channel weight, not the compensability exponent α of the main text); Cohen's d measures the effect size of deviating from 0.5; CI is the 95% bootstrap confidence interval for w^*. Cuba at $\texttt{mcs} = 50$ is omitted because HDBSCAN fails to produce a valid clustering ($K < 2$).

**Corpus**	`mcs`	K	w^*	CI	d
Cuba	3	31	0.45	[0.40, 0.45]	0.25
Cuba	5	14	0.50	[0.50, 0.50]	0.00
Cuba	8	12	0.45	[0.40, 0.55]	0.07
Cuba	12	2	0.55	[0.55, 0.60]	0.32
Cuba	20	4	0.55	[0.45, 0.55]	0.06
Cuba	30	2	0.55	[0.55, 0.60]	0.51
VisPub	3	41	0.50	[0.45, 0.55]	0.00
VisPub	5	21	0.45	[0.45, 0.50]	0.08
VisPub	8	11	0.50	[0.45, 0.55]	0.00
VisPub	12	2	0.50	[0.45, 0.60]	0.00
VisPub	20	2	0.50	[0.50, 0.65]	0.00
VisPub	30	2	0.55	[0.55, 0.60]	0.28
VisPub	50	2	0.60	[0.55, 0.65]	0.42

The Spearman correlation $\rho(K, w^*) = -0.85$ on Cuba ($p = 0.034$) and -0.61 on VisPub ($p = 0.147$) confirms that cluster count is the dominant sweep parameter.

3. Embedding-Model Sensitivity

The cross-corpus validation of the main text (Experiment 4) uses different corpora with a single embedding model each. To disentangle the effect of the embedding model from the corpus, we re-embed both the Cuba ($n = 418$) and VisPub ($n = 3{,}549$) corpora using two additional models, MiniLM-L6 ($d = 384$) and MPNet ($d = 768$), and recompute the full pipeline (UMAP→HDBSCAN→coherence) for each. Table S3 reports the key metric-level properties across all three embedding models on both corpora.

Table S3. Embedding model sensitivity on Cuba and VisPub. The metric-level properties (GM rank correlation with $d_\times$, triangle inequality violations) are stable across three embedding models of different dimensionality and architecture. K is the per-config cluster count (an emergent output of HDBSCAN, not a chosen value). Bracketed values are 95% bootstrap confidence intervals on $\rho_{A,T}$ from $B = 200$ document-level resampling iterations (seed 42, excluding same-document self-pairs); ρ_{GM} half-widths are within ± 0.002 of the point estimates. Triangle violation counts are out of 10^5 sampled triplets; the Wilson 95% rate intervals are reported separately below.

**Corpus**	**Embedding**	d	K	ρ_{GM}	Viol. (%)	$\rho_{A,T}$	(95% CI)
Cuba	GPT-4 (original)	1536	11	0.999	0.000	0.283	[0.231, 0.337]
Cuba	MiniLM-L6	384	12	0.999	0.000	0.294	[0.223, 0.355]
Cuba	MPNet	768	18	0.998	0.000	0.232	[0.174, 0.290]
VisPub	GPT-4 (original)	1536	113	0.992	0.000	0.234	[0.214, 0.255]
VisPub	MiniLM-L6	384	100	0.990	0.000	0.263	[0.240, 0.281]
VisPub	MPNet	768	101	0.990	0.000	0.267	[0.246, 0.290]

Wilson 95% intervals for the triangle-violation rates: all six configurations [0.000, 0.004]% (0 violations in 10^5 sampled triplets).

Takeaway. The GM rank correlation with $d_\times$ and the triangle inequality violations are stable across embedding architectures; only the channel correlation $\rho_{A,T}$ varies, and it stays in the low-correlation regime expected by scale complementarity.

GM rank correlation with $d_\times$ remains $\rho \geq 0.99$ across all six corpus-embedding combinations, and the triangle-inequality violation rate of $1 - C_{\mathrm{GM}}$ is 0% in every case (0 of 10^5 sampled triplets; Wilson 95% upper bound 0.004%). Every re-embedding clusters well above the degenerate regime (K ranging from 11 to 113), so the only substantial violation rate (7.6%) remains that of the degenerate $K = 2$ COVID configuration. Channel correlation varies across configurations ($\rho_{A,T} \in [0.232, 0.294]$) but remains low in all cases, consistent with scale complementarity across embedding architectures. The highest value ($\rho = 0.294$ for Cuba with MiniLM-L6) still corresponds to less than 9% shared variance ($\rho^2 = 0.086$), indicating that even with smaller embedding dimensions the channels remain non-redundant. The framework does not require low channel correlation, so this variation across embeddings does not affect the validity of the geometric-mean construction. The Max combinator remains the clear outlier ($\rho = 0.46$ to 0.62), confirming that its failure to track the product geometry is not an artifact of a particular embedding space.

4. Component Ablation

The combinator comparison of the main text (Experiment 2) ablates the choice of combinator. This section ablates two further design choices that the framework relies on, holding the Cuba GPT-4 corpus and the angular channel fixed:

- **(C1) Soft vs. hard cluster membership.** We replace the soft HDBSCAN membership vectors with their hard (one-hot) counterparts, assigning each document entirely to its most probable cluster.
- **(C2) Jensen-Shannon distance vs. divergence.** We replace the topic dissimilarity $d_{\mathrm{JS}} = \sqrt{\mathrm{JSD}}$ with the divergence JSD itself.

For each variant we recompute the Fisher identity correlation R_{Fisher}, the GM rank correlation with the product metric ρ_{GM}, the triangle-inequality violation rate of $1 - C_{\mathrm{GM}}$ on 10^5 sampled triplets, and the mean base-2 Shannon entropy of the membership vectors. Table S4 reports the results.

Table S4. Component ablation on Cuba GPT-4 ($n = 418$, $K = 11$). The baseline is the framework as specified in the main text (soft membership, Jensen-Shannon distance). C1 hardens the membership vectors; C2 substitutes the Jensen-Shannon divergence for the distance. R_{Fisher} is degenerate under hard membership: the Fisher-Rao distance collapses to a binary signal at the simplex vertices, so the Fisher-identity correlation is not meaningful (rather than literally undefined).

**Variant**	R_{Fisher}	ρ_{GM}	Viol. (%)	$\bar{H}$ (bits)
Baseline (soft membership, d_{JS})	0.994	0.999	0.00	1.92
C1: hard one-hot membership	—	0.650	0.00	0.00
C2: JSD divergence instead of d_{JS}	0.990	0.998	7.49	1.92

Takeaway. Both design choices are load-bearing. Hardening the membership vectors collapses the GM rank correlation with the product metric from 0.999 to 0.650; using the Jensen-Shannon divergence in place of the distance raises the triangle-inequality violation rate from 0% to 7.5%.

C1: soft membership. Hard one-hot assignment drives the mean membership entropy to exactly 0 bits: every document sits at a simplex vertex. This is the regime where the Fisher-Rao metric tensor degenerates ($1/p_k \to \infty$ as $p_k \to 0$), so R_{Fisher} is degenerate rather than informative. The topic similarity collapses to a binary same-cluster / different-cluster signal, and the rank correlation of C_{GM} with the product metric $d_\times$ falls from 0.999 to 0.650. The soft membership of the main pipeline is therefore not a cosmetic choice: it is what places the topic channel in the interior of the statistical manifold where the product-manifold interpretation holds. The 0% triangle-violation rate under one-hot membership is an empirical rather than a forced property: a cross-cluster pair has $d_{\mathrm{JS}} = 1$ and hence $D_{\mathrm{GM}} = 1$,

so any triangle containing a cross-cluster edge satisfies the inequality automatically, and only same-cluster triples can violate it—whether they do is a corpus-contingent property of the angular channel, not a theoretical guarantee.

C2: Jensen-Shannon distance. Substituting the divergence JSD for the distance $d_{\mathrm{JS}} = \sqrt{\mathrm{JSD}}$ leaves the rank correlations almost unchanged (ρ_{GM} from 0.999 to 0.998), because squaring is a monotone transformation and rank-based summaries are nearly insensitive to it. The metric structure, however, is not: JSD is not a metric (only its square root is), so the triangle-inequality violation rate of $1 - C_{\mathrm{GM}}$ rises from 0% to 7.5%. This is the empirical counterpart of the design argument in the main text for using the Jensen-Shannon *distance*: the distance is what makes the topic channel a proper metric and keeps the product metric $d_\times$ well-defined.

5. Per-Seed Embedding-Perturbation Results

This section complements Experiment 6 of the main text. Table S5 reports the per-seed values of K, R_{Fisher}, ρ_{GM}, the stability $\rho_{\mathrm{stab}}(C_{\mathrm{GM}}^{\mathrm{noisy}}, C_{\mathrm{GM}}^{\mathrm{clean}})$, and the triangle-inequality violation rate for $1 - C_{\mathrm{GM}}$ on Cuba GPT-4 ($n = 418$). The fixed-K block is deterministic up to the precision of the floating-point pipeline: the angular-channel noise leaves the rank ordering of C_{GM} exactly preserved at all three magnitudes. The floating-K block exposes the clustering instability discussed in the main text: at $\sigma \in \{0.01, 0.05\}$ one of three seeds collapses the cluster count to $K = 2$, the regime where the topic channel degenerates to a binary signal; the other seeds produce K in the range 13 to 16, close to the clean $K = 11$.

Table S5. Per-seed embedding-perturbation results on Cuba GPT-4 ($n = 418$). Three seeds per cell. Clean reference: $K = 11$, $R_{\mathrm{Fisher}} = 0.994$, $\rho_{\mathrm{GM}} = 0.999$, $\rho_{A,T} = 0.283$, 0.00% triangle violations. The final column gives the per-seed channel correlation $\rho_{A,T}$ whose seed means are reported for Experiment 6 in the main text. The fixed-K angular-ranking stability—$\mathrm{stab}(A) = 0.999995, 0.999886, 0.999543$ at $\sigma = 0.01, 0.05, 0.10$ (mean over seeds)—confirms the perturbation reaches the angular channel even though the C_{GM} ranking is held at stab $= 1.0000$.

Mode	σ	seed	K	R_{Fisher}	ρ_{GM}	stab C_{GM}	Viol. (%)	$\rho_{A,T}$
fixed-K	0.01	42	11	0.9943	0.9988	1.0000	0.000	0.2826
fixed-K	0.01	43	11	0.9943	0.9988	1.0000	0.000	0.2826
fixed-K	0.01	44	11	0.9943	0.9988	1.0000	0.000	0.2826
fixed-K	0.05	42	11	0.9943	0.9988	1.0000	0.000	0.2824
fixed-K	0.05	43	11	0.9943	0.9988	1.0000	0.000	0.2822
fixed-K	0.05	44	11	0.9943	0.9988	1.0000	0.000	0.2826
fixed-K	0.10	42	11	0.9943	0.9988	1.0000	0.000	0.2821
fixed-K	0.10	43	11	0.9943	0.9988	1.0000	0.000	0.2817
fixed-K	0.10	44	11	0.9943	0.9988	1.0000	0.000	0.2824
floating-K	0.01	42	16	0.9954	0.9986	0.6003	0.001	0.2553
floating-K	0.01	43	2	0.9970	0.9555	0.2788	0.534	0.1163
floating-K	0.01	44	15	0.9955	0.9983	0.6599	0.006	0.2985
floating-K	0.05	42	2	0.9963	0.9626	0.2380	0.563	0.0896
floating-K	0.05	43	14	0.9952	0.9989	0.6210	0.001	0.2440
floating-K	0.05	44	14	0.9950	0.9987	0.6039	0.003	0.2585
floating-K	0.10	42	13	0.9951	0.9984	0.6306	0.001	0.2958
floating-K	0.10	43	14	0.9952	0.9987	0.6082	0.000	0.3293
floating-K	0.10	44	16	0.9958	0.9979	0.5908	0.004	0.2891

The pattern is consistent with the discussion in the main text: where K stays near the clean value, all metric-level quantities are close to the clean reference; the only seeds that produce visible degradation in ρ_{GM} are the two cases where the perturbation pushes HDBSCAN into the $K = 2$ regime, and these are isolated to the smaller noise magnitudes $\sigma \in \{0.01, 0.05\}$ where the clustering boundary apparently sits closer to the clean configuration. These same two seeds also carry the lowest channel correlation ($\rho_{A,T} = 0.116$

at $\sigma = 0.01$ and 0.090 at $\sigma = 0.05$, the degenerate two-cluster value), so the floating-K seed-mean $\rho_{A,T}$ reported in the main text (Experiment 6) dips at those two magnitudes and is highest at $\sigma = 0.10$—the only magnitude at which no seed collapses to $K = 2$. The framework's robustness statement is therefore conditional: it holds at the metric level, given a stable clustering.

6. A Heuristic Rate-Distortion Analogy for the Geometric Mean

This section records the rate-distortion analogy referenced in the main text's case for the geometric mean (Section 4, on its log-additivity and scale-invariance properties). It is a heuristic information-theoretic reading of the log-additive cost structure, not a derivation: the geometric mean is already characterized in the main text as the unique combinator consistent with the four stated axioms, and the analogy below merely offers a parallel information-theoretic motivation for the same conclusion.

Suppose, hypothetically, that the transition from event d_i to event d_j is modeled as the joint transmission of two source components, one carrying semantic content and one carrying topical structure, with two simplifying assumptions:

(i) The two source components are independent, so the total rate decomposes as $R = R_A + R_T$.

(ii) The per-component rate-distortion functions take the logarithmic form $R_c(D_c) = -\log D_c + \text{const}$.

Under these assumptions, the joint rate-distortion function is $R(D_A, D_T) = R_A(D_A) + R_T(D_T)$, and the iso-rate curves in (D_A, D_T) space are level sets of $-\log D_A - \log D_T = \text{const}$, i.e., $D_A \cdot D_T = \text{const}$. A scalar summary of the distortion along such a curve must therefore be a function of $D_A \cdot D_T$ alone, and the choice $D = (D_A \cdot D_T)^{1/2}$ is the unique such summary satisfying the calibration $D = a$ when $D_A = D_T = a$, which is the same calibration as the normalization axiom of the main text. Because A and T are *similarities*, the distortion variable D_c here is fidelity-like and $R_c(D_c) = -\log D_c$ is *decreasing* in it: the analogy has the form of the Gaussian rate-distortion curve with the distortion axis reversed.

Neither assumption is a factual claim about the real channels. Assumption (i) requires strict statistical independence, which is structurally impossible in our setting because the topic channel is a deterministic function of the embedding channel via the UMAP→HDBSCAN pipeline. Scale complementarity shows that the channels are nevertheless *approximately* independent in practice (NMI = 0.027), and this is the only sense in which we use Assumption (i). Assumption (ii) is motivated by consistency with the log-additive cost structure rather than by first principles. The exact log-additive identity $-\log C_{\text{GM}} = -\frac{1}{2}\log A - \frac{1}{2}\log T$ holds at any correlation level and at any choice of underlying distributions, so the analogy above is best read as a conceptual motivation for the log-additive structure rather than as an independent argument for the geometric mean. The primary justification for the geometric mean in the main text is the axiomatic characterization.

7. Bootstrap Uncertainty for the Bottleneck-Gap Profile

This section complements Experiment 7 of the main text. The bottleneck-gap profile is validated by replication across five corpora; within each corpus we additionally quantify sampling uncertainty with a path-level bootstrap: the paired endpoint-pair index is resampled with replacement ($B = 2{,}000$), and on each resample we recompute the gap at the geometric mean $g(0)$, the Taylor coefficients $g'(0)$ and $g''(0)$, the leading-order predicted peak $\alpha^*_{\text{pred}} = -g'(0)/g''(0)$, and the observed argmax α^*_{obs}. Table S6 reports the 2.5/97.5 percentile intervals for the gap $g(0)$, its first two Taylor coefficients, and the observed peak on all four narrative corpora (Wikispeedia, with 10,832 human paths, is by far the largest

sample and its point estimates are correspondingly the tightest; it is omitted here only because the bootstrap was run on the four narrative corpora). On every corpus both $g(0) > 0$ and $g''(0) < 0$ hold across the entire 95% interval, so "the narrative trail is more coherent than random" and "the profile is concave at the geometric mean, hence has an interior maximum" are not point-estimate artifacts. The smallest corpus, COVID (40 documents, 169 endpoint pairs), is the only one where the magnitudes are loosely determined, and we detail it in Table S7.

Table S6. Path-level bootstrap ($B = 2{,}000$) of the bottleneck-gap profile on the four narrative corpora of Experiment 7. Intervals are 2.5/97.5 percentiles over the resamples; point estimates match the Experiment 7 table of the main text. The sign of $g(0)$ (trail more coherent than random) and of $g''(0)$ (concavity, hence an interior maximum) is stable across the full interval on every corpus.

Corpus	$g(0)$ CI	$g'(0)$ CI	$g''(0)$ CI	α^*_{obs} CI
Cuba	$[+0.518, +0.532]$	$[-0.072, -0.067]$	$[-0.078, -0.074]$	$[-3.0, -2.5]$
COVID	$[+0.159, +0.213]$	$[-0.008, -0.004]$	$[-0.009, -0.003]$	$[-6.0, -4.0]$
VisPub	$[+0.534, +0.551]$	$[-0.081, -0.074]$	$[-0.225, -0.212]$	$[-0.75, -0.5]$
AMiner	$[+0.542, +0.559]$	$[-0.083, -0.077]$	$[-0.243, -0.230]$	$[-0.5, -0.5]$

Table S7. Path-level bootstrap ($B = 2{,}000$) for the narrative-trail-versus-random bottleneck-gap profile on COVID ($n = 40$, 169 endpoint pairs), the smallest corpus of Experiment 7. Point estimates are those reported for COVID in the Experiment 7 table of the main text; intervals are 2.5/97.5 percentiles over the resamples.

Quantity	Point estimate	95% bootstrap CI
$g(0)$	0.186	$[0.159,\ 0.213]$
$g'(0)$	-0.0062	$[-0.0083,\ -0.0042]$
$g''(0)$	-0.0056	$[-0.0085,\ -0.0031]$
α^*_{pred}	-1.11	$[-2.20,\ -0.60]$
α^*_{obs}	-5.0	$[-6.0,\ -4.0]$

Every sign-level conclusion of Experiment 7 holds across the entire 95% interval, even on this smallest corpus: $g(0) > 0$ (the narrative trail is more coherent than a random chronological sequence), $g''(0) < 0$ (the profile is concave at the geometric mean and so has an interior maximum), and $g'(0) < 0$ together with $\alpha^*_{\text{obs}} < 0$ (the peak lies on the compensatory side of the geometric mean). At 169 endpoint pairs the *magnitudes* are only loosely determined—the observed peak is bracketed to $[-6, -4]$—but the qualitative structure is stable. On the three larger narrative corpora (418 to 6,000 documents) the same intervals are far tighter (Table S6); AMiner, the largest, has an observed peak of -0.5 in every one of the 2,000 resamples. The five-corpus replication reported in the main text is thus the principal robustness evidence, and the bootstrap confirms that the sign-level conclusions—$g(0) > 0$ and $g''(0) < 0$ on every corpus—survive within-corpus resampling, with only the smallest corpus showing wide magnitude intervals. The wide COVID interval is structural as well as statistical: with its small curvature $g''(0)$ the profile is nearly flat on the compensatory side, so the interior-critical-point guarantee of Proposition 5(c) lapses and the peak is weakly identified independently of sample size.

8. A Cross-Modal Case Study: Image Narratives (ROGER)

The five corpora of Experiment 7 are all textual. To probe whether the bottleneck-gap signature of the main text (the Proposition of Section 4.4) is specific to text or reflects the geometry of the composite metric itself, we apply the same analysis to a human-curated *image* narrative. We use the ROGER corpus of German et al. (*Semi-Supervised Image-Based Narrative Extraction: A Case Study with Historical Photographic Records*, ECIR 2025): 501

photographs of the 1928 Sacambaya Expedition (Robert Gerstmann Fonds, Universidad Católica del Norte), released with expert-curated baseline timelines—the same human-grounded resource and the same narrative-extraction task as Experiment 7, in a different modality.

Pipeline. The angular channel A is the cosine similarity of the repository's DETR ResNet-50 image embeddings, mapped to $1 - \arccos(\cdot)/\pi$ exactly as for the text corpora. The topic channel T is the UMAP→HDBSCAN soft membership on the *same* embeddings (the paper's own topic channel), with a fixed UMAP seed ($K = 20$ clusters). We deliberately do *not* build T from the corpus's expert theme labels: semi-supervised label propagation collapses those labels to a near one-hot membership, which sits at the simplex vertices where the Fisher–Rao tensor degenerates (cf. the component ablation of Section 4) and vetoes the theme-crossing transitions that a curated narrative is built from—it is not the soft simplex geometry the topic factor assumes. Images are ordered into a temporal DAG by their (label-propagated) expedition month, and storylines are forward paths in that DAG, as in the main-text bottleneck-gap analysis (Experiment 7).

Choice of storylines. The ROGER corpus provides its expert narrative at six nested lengths (5 to 30) because the original study compared against Narrative *Maps*, whose linear program lets one fix the extracted storyline to a target size and so match it to each expert length. Narrative *Trails* optimizes a different objective and returns the widest path whose length the graph dictates—it cannot be length-regulated—so the matched-length design no longer applies. We therefore adopt the natural alternative: extract the trail once and compare it against the expert baselines closest to its length. Here the trail has length 13 (bottleneck coherence 0.876, reliability 0.893), so we report the length-15 and length-10 expert timelines, keeping the human reference and the extracted trail of comparable size. This proximity criterion is independent of the outcome; as a bonus the two timelines have *distinct* bottleneck edges—the length-15 narrative's weakest link is a theme-crossing, channel-imbalanced transition ($A = 0.84$, $T = 0.07$), the length-10 narrative's is channel-balanced ($A = 0.84$, $T = 0.47$)—so they exercise two contrasting regimes of the metric.

Both expert storylines score above chance. Between the fixed (source, target) endpoints of each timeline we draw a one-sided Monte Carlo permutation null of $R = 5{,}000$ length-matched random forward sequences (the random count is the resolution of the null estimate, not a sample size). The expert timelines exceed their random null on every comparison—three of the four significant at 0.05, the length-15 bottleneck marginal ($p = 0.053$): at length 10, bottleneck coherence 0.629 versus a null mean of 0.180 (permutation $p = 0.021$) and reliability 0.760 versus 0.399 ($p = 0.007$); at length 15, bottleneck 0.243 versus 0.112 ($p = 0.053$) and reliability 0.675 versus 0.360 ($p = 0.025$). The extracted maximin trail scores higher still than either expert timeline, so the ordering is TRAIL > EXPERT > RANDOM; that the coherence-optimal extractor outscores the human curator is expected, since the expert curated for thematic and historical progression rather than for bottleneck coherence, and the load-bearing comparison is the non-circular EXPERT-versus-RANDOM one.

The bottleneck-gap signature transfers, and tracks channel balance. Sweeping the compensability exponent and decomposing each gap $g(\alpha)$ into its even and odd parts (the bottleneck-gap Proposition of the main text) reproduces the Experiment 7 pattern on both timelines: the even (symmetric) part is maximized at the geometric mean (swept argmax $\alpha = 0$; closed-form curvature $g''(0) < 0$), and the design-premise condition $\mathbb{E}_{\mathrm{R}}[Y_\alpha] \geq \mathbb{E}_{\mathrm{H}}[Y_\alpha]$ holds at every swept α. The two regimes differ in *how pronounced* the peak is, exactly as the channel balance of the bottleneck predicts. For the balanced-bottleneck length-10 narrative the peak is sharp ($g''(0) = -0.16$, leading-order peak $\alpha^* = -0.6$, even part down 22% by $|\alpha| = 1$); for the imbalanced-bottleneck length-15 narrative it is shallow

($g''(0) = -0.02$, $\alpha^* = +3.3$, even part down 60% by $|\alpha| = 1$ but very flat near the apex). Here the quadratic term alone predicts only an $\approx 8\%$ drop by $|\alpha| = 1$; the remaining decline is carried by higher-order even terms, large because the bottleneck imbalance ($\Delta \approx 2.5$) shrinks the Taylor radius, so the near-flatness holds only for $|\alpha| \lesssim 0.3$. The geometric mean is the symmetric optimum in both cases; the strength of that optimum scales with how balanced the narrative's weakest transition is.

Scope. ROGER provides a single expert narrative, so the two reported lengths overlap and are not statistically independent; this is an illustrative cross-modal case study, not a powered population test like Wikispeedia (10,832 independent human paths). It is reported as corroboration that the even-part-at-the-geometric-mean structure is a property of the composite metric rather than an artifact of the text corpora. As noted above, the TRAIL–EXPERT comparison is between fixed endpoints rather than at matched length, since Narrative Trails cannot be length-regulated.

9. The Veto under Cardinality-Constrained Extraction

The veto property of the geometric mean—$C_{\mathrm{GM}} = \sqrt{A \cdot T} = 0$ whenever $T = 0$, i.e. a transition between disjoint topic clusters—is, by the combinator-selection argument of the main text (Discussion), redundant for unconstrained maximin (Narrative Trails) extraction but load-bearing under the cardinality-constrained linear program of Narrative Maps. This section quantifies that contrast.

Setup. On the Cuba corpus ($n = 418$, canonical $K = 11$ soft clustering) we draw 30 source–target pairs (source in the first temporal third, target in the last; seed 42) and extract a storyline for each under two regimes and three combinators: the geometric mean C_{GM}, the product-metric complement $1 - d_\times$, and the arithmetic mean C_{AM}. The first regime is the maximin (widest-path) extractor of Narrative Trails; the second is the Narrative Maps linear program, whose *cardinality* constraint $\sum_i \text{node\_act}_i = K_{\mathrm{LP}}$ fixes the storyline to K_{LP} active nodes—the "expected-length" constraint of the Maps LP, and *not* its separate topic-coverage constraint (see the caveats). We sweep K_{LP} from 6 to 25. The veto acts at graph construction: edges are instantiated only where coherence is positive, and since $C_{\mathrm{GM}} = 0$ on every disjoint-topic edge, all 6,203 of the 87,153 document pairs with $T = 0$ are absent from the geometric mean's graph. We report how many of the 30 extracted storylines contain an edge with $T = 0$ exactly—the veto's precise boundary.

Maximin: the veto is redundant. Under the unconstrained widest-path extractor, *all three* combinators avoid disjoint-topic edges entirely (0/30 each, Table S8): the bottleneck objective discards the lowest-coherence edges regardless of combinator, so the veto adds nothing. This is the "redundant for maximin" half of the claim, and it holds for the veto-less combinators ($1 - d_\times$, C_{AM}) as well.

Cardinality-constrained LP: the veto is load-bearing. Table S8 reports the sweep. The geometric mean traverses a disjoint-topic edge in 0 of 30 storylines at *every* length—a structural guarantee, since its graph contains no such edge—whereas the veto-less combinators are routed through disjoint transitions at a rate that *grows with storyline length*, from 1–4 of 30 at $K_{\mathrm{LP}} = 6$ to 15–18 of 30 (half or more) at $K_{\mathrm{LP}} = 25$. The cardinality requirement forces a longer chain; without the veto that chain is increasingly routed onto disjoint edges, whereas the geometric mean's pruned graph cannot supply one. The result is deterministic for a fixed pipeline—the flow-pruned extraction and the linear-programming solver are both deterministic—so the only source of run-to-run variability is the upstream UMAP→HDBSCAN clustering, characterized in Section 5 and Experiment 6 of the main text.

Caveats. Two points fix the scope. (i) *Cardinality, not coverage.* The swept constraint is the Maps LP's node-count constraint, which sets storyline size; it is *not* the LP's separate

Table S8. Veto under cardinality-constrained extraction (Cuba, $n = 418$, $K = 11$, 30 endpoint pairs, seed 42). Each cell is the number of extracted storylines containing an edge with $T = 0$ exactly (a disjoint-topic transition; the veto boundary). Maximin is length-invariant; the linear program is swept over its cardinality (storyline-size) constraint K_{LP}. The geometric mean is 0/30 at every length because $C_{\mathrm{GM}} = 0$ prunes disjoint edges from its graph, while the veto-less combinators traverse them at a rate rising with length.

**Extractor**	C_{GM}	$1 - d_\times$	C_{AM}
Maximin (Trails), any length	0/30	0/30	0/30
LP, $K_{\mathrm{LP}} = 6$	0/30	1/30	4/30
LP, $K_{\mathrm{LP}} = 8$	0/30	5/30	5/30
LP, $K_{\mathrm{LP}} = 10$	0/30	6/30	6/30
LP, $K_{\mathrm{LP}} = 12$	0/30	11/30	8/30
LP, $K_{\mathrm{LP}} = 15$	0/30	10/30	12/30
LP, $K_{\mathrm{LP}} = 20$	0/30	14/30	14/30
LP, $K_{\mathrm{LP}} = 25$	0/30	15/30	18/30

topic-coverage constraint. We separately implemented the actual coverage constraint (the Maps "MinCover" requirement, whose per-cluster credit is the Bhattacharyya coefficient $\sum_k \sqrt{\hat{e}_{ik}\,\hat{e}_{jk}}$ of the endpoints' membership vectors) and swept its threshold: because that credit rewards within-cluster (high-T) edges and assigns disjoint-topic edges essentially zero credit, coverage steers the path *toward* topical cohesion and only reduces disjoint-edge traversal. The cardinality-only result reported here is therefore conservative—adding coverage strengthens, not weakens, the veto's relevance. (ii) *The metric is the exact boundary* $T = 0$. The veto is the statement $C_{\mathrm{GM}} = 0 \iff T = 0$; relaxing the threshold to $T < 0.05$ admits *near*-disjoint edges ($0 < T < 0.05$, where $C_{\mathrm{GM}} = \sqrt{A \cdot T} > 0$ and the veto does not act), on which the geometric mean is small but nonzero (0 to 6 of 30 across the sweep). $T = 0$ is thus the faithful metric for the veto, and it is there that the geometric mean is uniformly 0.
